# “I Live Quite a Good Balanced Life”: A Pilot Study on the Life Experiences of Ageing Individuals Living with HIV

**DOI:** 10.1155/2012/128108

**Published:** 2012-06-17

**Authors:** Nuno Ribeiro Nobre, Jari Kylmä, Tapio Kirsi

**Affiliations:** School of Health Sciences, Public Health, University of Tampere, 33014 Tampere, Finland

## Abstract

An ageing population is accompanied by an increased number of older adults living with HIV. So far, our knowledge regarding the life experiences of older adults living with HIV is still poor and under researched. The purpose of this study is to present new knowledge by interviewing nine Finnish HIV-positive individuals of 50 years of age and older. The data were analysed by inductive content analysis. Living with HIV is shaped by unique personal life experiences. These experiences played an important role on how the interviewees assessed their lives and their future as HIV positive individuals. Most of the participants reacted negatively to their HIV-positive diagnosis. However, throughout time they had found meaning in their lives and had developed a degree of positive attitude towards life and future which was articulated in terms of a good overall balanced life. Since caring is the tenor of the nursing profession, nurses should be able to identify and implement methods for assessing how successfully older adults living with HIV age and intervene in an informed way whenever needed.

## 1. Introduction

The number of older adults living with HIV is increasing yearly. National Institute for Health and Welfare (THL) [1] surveillance data estimated that by the end of November 2011, 346 individuals aged 50 and older were living with HIV in Finland. This relatively small number of older adults living with HIV will tend to increase in years to come, not only because the population living with HIV gets older but also because the number of new transmissions is increasing among this age group. So far, there is no research-based knowledge regarding to this section of the ageing Finnish population. This study is the first attempt to explore the life experiences of 50+ people living with HIV in Finland. The classification of study participants by age is based on the CDC's age categorization system, widely used in scientific literature [2]. 

Older adults living with HIV have been largely invisible in the field of HIV research. They rarely participate in study projects, and when they are included, age is not used as a central variable in the analysis of the data [3]. HIV among older adults is also a neglected issue among health care professionals. On the one hand, these problems entail misdiagnoses as a result of doctors failing to suggest HIV tests to the patients. On the other hand, there are difficulties in recognizing a range of symptoms actually related to the HIV rather than to ageing itself [4–6]. 

Becoming HIV positive is a life changing event [7, 8]. Receiving a positive HIV diagnosis raises two crucial questions: how is the news delivered and how supportive or judgmental are the health professionals? [9]. These two questions may play an important role in how individuals react and learn to cope with their diagnosis. Following an HIV positive diagnosis, individuals' coping mechanisms may be challenged at several levels.

The ways how individuals react to their positive HIV diagnosis vary. Previous studies reported shock as the most frequent reaction [8–11]. Recovery from such reaction is interrelated to internal and external factors. The duration of the period of initial shock amongst older patients is rarely addressed in the existing literature on the topic. 

Sociological studies characterize shock as two types of reactions: emotional and behavioral. Emotional reactions involve states such as fear, shame, depression and suicidal thoughts [9, 11]. Behavioral reactions include actions taken by individuals like misuse of alcohol and illicit drugs [12]. 

The existing research on older adults living with HIV has focused mostly on individuals' emotional reactions. The findings of these studies have suggested that after being diagnosed with HIV, older patients tend to go through a process of introspection. This introspection enables individuals to find meaning in their lives as an HIV positive. In previous studies searching for meaning of life has been seen as a coping mechanism which raises hope and helps to avoid feelings of despair, often linked to drastic life changing events [13–16].

Hope is a self-continuous process of finding meaning in life, empowering individuals to deal with actual and upcoming negative events. In previous studies ways of finding hope in life have been reported to be associated with levels of social support and religion. Additionally, hope is a source of self-worth in awakening the connection between self and others in a harmonious way, significantly associated with Quality of Life (QoL) and successful ageing with HIV [17, 18]. 

The findings of the few qualitative studies on older adults living with HIV have shown that growing older with HIV may entail personal advantages which are essential for maintaining life satisfaction and QoL. These advantages include factors like self-acceptance, wisdom and positive attitude towards life. [2, 19, 20]. Acknowledging such sources of strength in the older population offers a good baseline for health and social care interventions aimed at promoting healthy ageing with HIV.

## 2. Purpose of the Study 

The purpose of this study is to explore the experiences of Finnish older adults living with HIV since the time of diagnosis until the time of being interviewed. 

## 3. Participants

To collect the data for this study an HIV support group located in Helsinki was contacted. After giving his permission to conduct the study the leader of the support group contacted the group members by phone and asked about their willingness to participate in the study. Eight interviewees were recruited from the support group. One interviewee was an acquaintance of the first author of this paper. 

The data of the study consist of nine persons, seven men and two women. The inclusion criterion was being 50 years old and older and HIV positive.

The age of the participants ranged from 51 to 73 years, with the mean age of 60.6 years. Three participants were infected with the HIV at the age of 50 years or older. The number of years living with HIV ranged from 4 to 24 years, with the mean years of 11.1. Eight participants were in the asymptomatic HIV phase and one in the AIDS phase. Most of the participants had an educational background equivalent to or higher than high school degree. In this sample homosexual contact was the major route of transmission of HIV infection (*n *= 5). Most of the participants were divorced (*n *= 5). For all of them divorce has occurred due to personal reasons other than HIV infection.

## 4. Data Collection and Procedure

The data were collected by using an in-depth interview technique in which the participants were asked to tell about their lives in their own words by responding to the following question: “Could you describe your life experience after you were diagnosed as HIV-positive until today?” Further questions were posed depending upon the course and the content of each of the interviews. This technique allowed the interviewer to question in-depth the life experiences of the participants. The interviews were conducted by the first author of this paper between June 2010 and July 2010 and lasted from 30 minutes to one and a half hours.

The interviews were conducted at different locations chosen by the participants. Eight interviews were carried out in the facilities of the support group, one at the researcher's home and one at the University of Tampere. 

## 5. Method of Analysis

Each interview was *verbatim *transcribed in Finnish. In order to correct possible inaccuracies, the written transcripts were reviewed by the researcher by replaying the recorded interviews at the same time as reading the written documents. 

The interviews were conducted and analysed in Finnish language and only the extracts referred in the study were translated into English. The translation of the extracts was checked by the authors for accuracy and consistence. Only the first author is aware the of participants' identities; hence the anonymity of the interviewees was secured throughout the entire process of the study. Initially, the transcripts were read and reread and replayed in order to familiarize the researcher with the contents of the interviews. After this first stage, a detailed analysis of the contents was performed. This detailed analysis included data reduction (coding), grouping (categorization), and finally the abstraction of ideas and concepts raised from the data [21]. During careful reading of the transcripts, all the statements (words and sentences) that were related to each other were marked with different colours. Reduced expressions were derived from the original statements, which in turn were grouped as different subcategories. Subsequently, subcategories were organized into broader categories under the main theme of experiences of living with HIV.

The data were analysed qualitatively by using inductive content analysis method. Since life experiences of older adults living with HIV have not been researched in Finland, the use of inductive content analysis as the method for analysing the interviews was considered an appropriate one [22]. 

## 6. Findings 

The analysis of the participants' narrations produced three main categories: individual reactions to the HIV diagnosis, the impact of HIV infection on daily life, and a good balanced life as a 50+ HIV positive person.

### 6.1. Individual Reactions to the HIV Diagnosis

The interviewees' talk of their individual reactions to HIV diagnosis was talk of shock, thoughts about death, fear of others becoming aware of their HIV status, use of alcohol, and no shock. Seven participants reported experiencing feelings of shock after acknowledging their HIV positiveness:

* when I got to know that I was HIV positive I went into shock...In the beginning it was horrible, I was in the hospital for one week, I was in a deep shock, I got fever up to 40 degrees. (Woman 2) *

The length of shock depended on individual's personal strength and on having someone to talk with:

*...in the beginning it was such a shock, a kind of shock phase...however it passed pretty quickly...Well of course, it's the first shock, such a shock that made me to think about life and it felt that, it all came at once and I needed to release the stress over me...as soon I got to know about the results [HIV diagnosis] I told a close friend. He was my huge mental support in the beginning. I think if I would not have had anybody to rely on, the shock phase would have been longer. (Man 7)*

Inappropriate information during the 1990s was expressed as a source of thoughts about death by three participants:

*...in the beginning information was rather fragmented in a way, and...and it was pretty...pretty like...well I thought that I will die straight away.... (Man 6) *

Associated with this lack of information is the fear of others becoming aware of the participants' HIV status. In the beginning of the HIV epidemic, HIV was associated with distressing and horrific images of people in the advanced stages of AIDS, which impacted and influenced how infected individuals thought about the disease:

*...in a way it was in the beginning, I thought in a way that it could be seen on me somehow...in the beginning it was such a horrible feeling, it felt that somehow people could see it. (Man 6)*

Three of the interviewees reported that getting the HIV positive diagnosis had led them to excessive use of alcohol as an escape from the reality. This behavioural reaction may imply incapacity to deal with life stressors in a reasonable manner. One participant described it as:

*well I started to drink after my good friend's death; it was a bad moment in my life...Honestly I must say that my drinking increased after I was diagnosed...At the moment I'm drinking much again because I'm quite worried about my brother-in-law's health...He needs daily support and I'm the one who takes care of him.... (Man 5)*

Interestingly, three participants described their reaction to their HIV-positive diagnosis as experiencing no shock at all. Past experiences with HIV, belonging to a risk group or being under cancer treatment, all played a role in their neutral reaction. This was described by the participants in the following way:

*I didn't get any kind of shock. I lived in Africa, Zimbabwe, for some years. I went there for teaching duties...I saw many people dying every day. It was a familiar thing to me. There I saw [HIV] before I got infected therefore it was a really familiar situation to me. A couple of friends got infected there also. I didn't make a complex thing out of it [HIV]. It [HIV] didn't affect my life in any way. (Woman 1) *

*...well it was not nice but I was prepared for it...I went without any referral to take a test [HIV test] because I heard about the disease and it was clear that I belonged to a risk group.... (Man 3) *

*...then it came this HIV, I thought that is one more [disease], however it is this cancer that will determine what will happen in my life. In my mind, I really thought about these things.... (Man 1). *

The previous extracts from those three interviewees expressed a life-course perspective based on the ability of some individuals to absorb past life experiences and transform them into practical events of life, enabling them to overcome successfully with upcoming obstacles.

### 6.2. Impact of HIV Infection on Daily Life

Most of participants expressed a strong belief that living with HIV for many years can become a positive element in one's lives. This was linked to the personal meaning of HIV and individual life changes after HIV diagnosis.

#### 6.2.1. Personal Meaning of HIV

Living for years with HIV had redirected some of the participants' attention from the life-threatening disease to life itself. This attitude was described in terms of seeing HIV as a normal chronic disease rather than a terminal illness. One participant strongly expressed his personal view on his HIV infection

*“...HIV is not all me, it is only a small part of me. Inside of me there is much more than HIV” (Man 2) *

while another participant simply stated:

*“...regarding my future, well, I take into account ageing issues rather than HIV issues” (Man 6). *

Both expressed a positive outlook towards growing old as an HIV positive. 

The statements of three participants illustrate how not dwelling on their HIV positive diagnosis enabled them to lead a normal life as anybody else:

*“...I don't have any reason to blame [HIV]...the world is full of all kind of diseases...” (Woman 1). *

Similarly, another participant referred to his personal ability to deal with life stress and his lack of information about HIV virus transmission:

*...well how I could say it. HIV is not guilty of anything. It's a fate, illness is a fate...is fate against it one can't do nothing about it...is a fate...it didn't fall from heaven, I have acquired it. Of course earlier on there wasn't enough knowledge [about HIV] so I can't even say that it was stupidity from me...before being infected I didn't have any source of knowledge about the disease's existence. (Man 3)*

The two insights cited previously into HIV along with its manageability due to advances in medical research illustrate the way most of the participants faced HIV as a normal chronic disease among others. One participant expressed it in the following way:

*...it is a treatable disease, same as others diseases...does it really matter if you have diabetes or HIV...it is the same, do I have HIV or other disease, I had cancer before and the same way I face HIV.... (Man 3)*

#### 6.2.2. Individual Life Changes after the HIV Diagnosis

Several participants brought out positive effects of HIV in their lives. One of them put it:

*...it is a funny thing, it has been a very positive thing for me. People should believe it, it has brought many positive things.... (Woman 1)*

Narratives of becoming braver, of life enrichment, of becoming religious, and of the benefits of retirement arose from the interviews. One female participant described how HIV has made her a stronger person:

*...I'm braver than before, I always have been brave but I feel now even braver than before.... (Woman 2)*

A male participant explained how religion had helped him to overcome his initial shock:

*...God? Yes I've [getting religious]...I prayed all the time to God and in certain way, searched, wanted to know or confront myself, I wanted to be with my own God face to face, this is based on a book by Gandhi.... (Man 4) *

One male participant expressed how early retirement has played a big role on his personal life:

*“...I decided to end my working life...I'm retired now...I have lived a very good life for 10–12 years with HIV infection, I have time for myself and for my family...” (Man 2)*

Sexual readjustment was mentioned by some participants as a positive aspect of being infected with HIV:

*...of course it [HIV] has changed the way I address others sexually. Nowadays I'm very careful, selective and reserved, above all I take others into account but at the same time also myself, those cross-infections are not good. I must be extremely careful.... (Man 7) *

On the other hand, also negative aspects of sexual readjustment were brought forth:

*...with this age sexual desire is in some way declining, and then this HIV... is in a certain way a barrier that stops me from swinging around.... (Man 6)*

Due to concerns about safety, masturbation was the only form of sexual satisfaction for one male participant:

*...there is a certain boundary in every sexual encounter. For example I won't come inside of anybody, even though knowing that my viral load is very low...At the same time I fear cross-infections. Well nowadays it is more just like self-masturbation. (Man 2)*

Interestingly, both the oldest and the youngest interviewee in the data stressed how their own life courses enabled them to take control over the situation by making use of their past experiences:

*...I didn't allow HIV to have an impact on my daily life...I have had my own hobbies for a long time and I still have them...no friendship has been broken, neither has any kind of friendship been formed due to HIV...the circle of friends that I have at the moment, it's the same as it has been even before HIV.... (Man 7) *

*...it didn't affect me at all [learning about her HIV positive diagnosis]... I'm a natural sciences teacher, diseases are a well know concept, accepting death as part of life is as natural as birth. Besides, I grew up in a hospital environment, my parents worked in a tuberculosis health care institution, therefore chronic diseases are familiar to me...I have also lived and worked in Zimbabwe...I saw people living and dying due to HIV infection...HIV hasn't affected my life.... (Woman 1) *

Both of these quotes express how people can maintain a positive outlook and try to avoid mental strain by acting rationally. 

#### 6.2.3. Good Balanced Life as a 50+ HIV Positive

The talk of balanced life as a 50+ HIV positive was linked to good normal life, optimism regarding the future, advantages of being HIV positive in later adulthood, mentoring others, and having a positive outlook to one's HIV infection. Most of the participants reported having a positive stance on ageing with HIV. Furthermore, they remained open about their prospective ability to enjoy life, maintaining and defining new future goals and being able to help others at an educational level regarding to HIV. The narrative of the present and future life events was put into words by two female participants:

*...quite normal. As normal as before. (Woman 1)*

and

*Well I face the future with hope, I really want...studying is perhaps at the moment the most important thing, but if I could get work in some kind of organization that would be great. Regarding love matters, I hope one day to find a man; it doesn't matter if he is positive or negative. (Woman 2)*

On the other hand, this positive outlook had enabled some participants to develop a good relationship with the disease. One female participant described how she regards HIV as a minor manageable issue in her life:

*...why should I make HIV a special problem, I have lived 13 years with it and so far I haven't had problems with it, why should I start now, no point in it...I have a good theoretical relationship with my HIV positivity. More theoretical than practical, in practice I don't have anything other than HIV medication. Is not even a big thing, I take those [medicines] as multi-vitamin tablets. (Woman 1) *

One male participant talked of his medication much in a similar way:

*...it is a good thing that I have three pills. In the morning I take them as if they were vitamin pills. (Man 5)*

Being infected at an older age was acknowledged by the participants as a central factor in their life in overcoming some of the negative aspects related to getting infected at younger age. This is illustrated by the comment of one female participant:

*I was over forty when I got to know that I was HIV positive...somehow I had time to mature as a person at that time...it was somehow easier to deal with the information...However I think it could be difficult to deal with [HIV] if I had been infected in my twenties or thirties...I had experienced almost everything, travelling a lot, well everything…. (Woman 2)*

Similarly, one male participant reinforced the positive aspect of being infected at older age by stating:

*...it was a big relief that I wasn't working anymore. Thank God I was retired and thank God I have seen the world...I wasn't afraid of anything, especially in the way how I should live. Back then I thought lucky me for being twenty years older when I got to know. (Man 5)*

While growing older, most people feel themselves responsible for advising and educating younger generations. This feeling was expressed by one female participant:

*...I teach...HIV has influenced me in a way that every Fall I reserve two hours of my teaching schedule to HIV, because HIV belongs to human biology.... (Woman 1)*

For one male participant mentoring others was seen as a self-rewarding experience:

*...I have been asked to give conferences on HIV as an infected individual and as a person who has passed different phases of the disease...I really like it, it is very rewarding giving others information about prevention and care...in my audiences there are also health professional students, I think it is important to show them what HIV is about...I also mentor others.... (Man 3)*

## 7. Discussion

Participating in in-depth interviews enabled nine HIV-positive older adults to verbalise and articulate their experiences as ageing individuals with HIV. This study provides knowledge base in the field of HIV research in two different ways. Firstly, this was the first study conducted in Finland on older adults living with HIV. Secondly, this study advances our understanding of the life experiences of older adults living with HIV and at the same time supports the small number of previous studies on the issue of life experiences as an HIV-positive person, particularly among older adults.

Most of the participants had reacted negatively to their HIV positive diagnosis by means of shock. This finding was in accordance with previous studies [8–11, 15, 16, 23]. In addition to shock, fear of disclosure of HIV status and thoughts about death were other negative reactions shared by most of the participants who were infected in the 1990s. These negative reactions were related at least partly to the lack of sound information and negative media discourses around HIV disease during the first years of the HIV epidemic. These findings coincide with those of Nichols et al. [24] on older adults infected with HIV in the 1990s. Similar to some respondents of the studies of Emlet et al. [2] and Skaggs and Barron [25], two participants from our study told that they had reacted neutrally to their positive HIV diagnosis. These reactions were associated with personal past experiences of people participating in the studies.

Learning about one's HIV diagnosis, had led two participants in our study to excessive use of alcohol. This behavioural reaction was also noted by Immonen et al. [26] who found that older people used alcohol as a way to alleviate their anxiety and depression. This type of behaviour, irrespective of its individual cause, can put older adults at risk of becoming alcoholics, especially if alcohol is used for relieving emotional problems [26, 27].

From the data of this study the duration of initial shock varied from some weeks to years. The duration of shock was somewhat longer amongst those participants who were infected in the 90's. For some participants the length of shock was associated with their inner need to cope with their changing health either individually or with the help of health professionals. On the other hand, those infected at the age of 50 or older attributed the relative shortness of their shock period to having someone to talk with and to their own personal strength. The findings from this study provide support to the concept of wholesome knowledge, according to which life experiences are used at older age to deal with present difficulties in a more rational way [28]. Therefore, ageing individuals may react differently to life changing events compared with their younger counterparts. However, it remains unclear whether age or having someone to talk with or both influence the length of shock.

Our analysis revealed that life changes after receiving the HIV positive diagnosis proved to be assessed more positively than negatively. Becoming braver and experiencing life enrichment were aspects mentioned by some participants. Barroso and Powell-Cope [17] and Nichols et al. [24] noted that those individuals who had found positive meanings in living with HIV/AIDS were usually more able to develop good coping mechanisms to deal with daily life changing events. Early retirement, for some of those who were infected with the HIV virus in the 1990s, was expressed as a positive life changing event. With early retirement individuals have more time for themselves and for their families and friends. These findings are in line with the social gerontological disengagement theory according to which, by getting older, individuals pass through a series of social disengagements [29]. The way losses and gains are balanced allow the individuals to sustain a high level of functioning and well-being [30].

In our study religion turned out to be one of the ways of coping with the disease. Barroso and Powell-Cope [17], Kremer and Ironson [31] and Ironson et al. [32] also noted an increase in religiousness after being diagnosed with HIV as a source of spiritual well-being and overall quality of life improvement. These findings suggest that the reasons found for younger HIV-infected adults for increased religiousness remain valid for older adults as well.

Readjustment of sexual behaviour as one aspect of life changes after being diagnosed HIV positive guided the way how sexual desires were expressed by the interviewees; the topic was addressed by talk of capability to carry on normal sexual activity without totally excluding sexual activity from ones' life. Most of the participants of our study had readjusted their sexual behaviour in a positive manner; this resembles what Emlet et al. [2] described in their study as an increased interest in the health and well-being of both the infected person themselves and their sexual partners. On the other hand, Baumgartner and David [16] and Maticka-Tyndale et al. [33] noted that individuals tend to suppress their sexual desire and opt to be in a state of celibacy after being diagnosed with HIV infection. Similarly, Maticka-Tyndale et al. [33] also suggested that individuals tend to find other ways to satisfy their sexual needs instead of sexual intercourse. 

For the majority of interviewees in our study living with HIV had enabled them to amass a wide range of new life experiences. Regardless of the nature of these experiences, the participants had assimilated them in their every day practices and used them to construct their identities, which were expressed as having a balanced life. For Baumgartner [15] and Baumgartner and David [16] this represents a so-called self-integration process which boosts individuals' self-esteem, and increases the sense of well-being and self-acceptance [2, 34], which help individuals to maintain or adopt new roles by means of engaging in meaningful social activities that in turn will influence their QoL and consequently successful ageing with HIV.

Closely associated with self-acceptance as an HIV-positive individual is the optimism towards life. Optimism as a means of finding meaning in life as HIV positive was described as a natural and a necessary means of self-development and as a better strategy for coping with the disease. Hopefulness was manifested as an ability to see the HIV as a small part of one's identity, reflecting a successful internal decentralization of HIV from self by means of reintegration in social life as an individual with a chronic disease rather than as an individual with HIV. These results reinforce the findings of Baumgartner [15] of 18 adults aged from 23 to 45 years living with HIV/AIDS who noted that *“the respondents realized that they were more than their HIV/AIDS identities”* [Ibid. 925]. Nichols et al. [24] and Kremer and Ironson [31] have also noted the minor significance that older people gave to HIV infection in their lives. 

Optimism was revealed to be an important coping mechanism reflecting a positive outlook regarding the present and future. This concept is in accordance with The Preventive-Corrective Proactivity (PCP) Model of Successful Ageing developed by E. Kahana and B. Kahana [35] which comprises a set of social and psychological factors that influence individuals' QoL and fosters positivity in life in face of illness. It also reinforces the Selection, Optimization, and Compensation (SOC) model developed by P. B. Baltes and M. M. Baltes [36] that suggests that successful ageing depends upon the ability to set, pursue, and maintain personal goals, associated with cultural and individual cognitive appraisals which are mediated by processes of selection, optimization and compensation. These processes enable ageing people to maintain positive thinking and functioning in spite of negative health changing events. The participants' responses revealed features of successful ageing proposed by these two models (e.g., optimism, normal life, mentor, good relationship with the HIV infection).

Positivity as a result of a continuous process of adaption to the disease is closely linked with the concept of resilience. Ong et al. [37] found that resilience refers to the individual's ability to cope with and recover positively from negative events in later adulthood. It is also coherent with the findings of Emlet et al. [2] who have noted that older people living with HIV have a strong will of living moderated by their resilience to adapt to negative life events. Siegel et al. [19] have characterized this as wisdom. Both terms, resilience and wisdom, refer to the ability to recognize strengths and limitations and take advantage of them in a positive and rational way. It is clear that being resilient has helped the informants to move ahead in their lives.

Being able to accept oneself as an HIV-positive individual has led to an inner desire to help others by means of education. Mentoring others is a result of constant personal growth resulted from a superior level of maturity and wisdom. Being both educators and advocators was expressed as an important component by some of the participants, giving a sense of personal reward. Siegel et al. [19] and Emlet et al. [2] also noted in their study the importance of generativity in older adults living with HIV/AIDS as a mode of educating others. 

## 8. Ethics

Ethical consent to carry out this study was granted by the leader of the support group. This support group is part of an independent association that offers psychosocial support to HIV-positive individuals and their families. The participation in and affiliation with the activities of the association is voluntary. The study was based on the articles 6, 11, 22, 23, and 33 of the Helsinki declaration [38] and on the Beauchamp and Childress [39] bioethical principles: autonomy, justice, beneficence, and nonmaleficence. The participants were informed by the leader of the support group and by the researcher about the purpose and content of the study, thus respecting the participants' autonomy and free participation by means of informed consent.

## 9. Credibility and Limitations of the Study

The credibility of the study was monitored by continuous check-ups since the beginning of the study by the research team, involving constant discussions of data collection, analysis, and reporting of the findings. 

However, there are some limitations that need to be acknowledged. First, the participants were recruited from a limited geographical (capital) area of Finland. Second, the sample size was rather small and the group quite homogenous. Women and heterosexual men may have different experiences of living with HIV than homosexual men, who formed the majority of our data. Thirdly, even though all the interviewees participated voluntarily in the study and most of them were members of the support group, it is possible that some older adults living with HIV and who have experienced or experience HIV in a more negative way, were not reached by the study. Given this, the transferability of the results is somewhat questionable because the findings are context-bound. However, even though it may not be possible to generalize these findings to other populations, they provide an insight into life experiences of older adults living with HIV and can offer useful information to health care institutions in developing future health and social care interventions adjusted to the needs of different segments of people living with HIV.

## 10. Conclusion

The results of this study provide an insight into older adults' experiences in living with HIV as a personal journey characterised by some challenges which were overcome by means of personal strengths and a strong will of living, marked by sources of resilience in coping with the disease. Researchers as well as health and social care providers need a better understanding about the factors that promote older adults living with HIV to cope with their disease as well as those that avert individuals from growing old successfully with HIV, thus improving the health and functioning of the infected older adults and their significant others. More research is needed in order to provide a good psychosocial support for those who are mostly forgotten. 
